# Neonatal stridor presents at home – vocal fold paralysis as rare presenting feature of CHARGE syndrome

**DOI:** 10.1515/crpm-2022-0033

**Published:** 2023-05-05

**Authors:** Sierra S. Donnell, Megan K. Kraemer, Suhagi M. Kadakia

**Affiliations:** Department of Pediatrics, Rush University Medical Center, Chicago, IL, USA; Department of Pediatrics, Division of Neonatology, Rush University Medical Center, Chicago, IL, USA; Division of Neonatology, Fetal Neonatal Medicine Center, Rush University Children’s Hospital, Chicago, IL, USA

**Keywords:** CHARGE syndrome, neonatal stridor, vocal fold paralysis

## Abstract

**Objectives:**

To present an unusual presentation and diagnosis of CHARGE syndrome with vocal fold paralysis, a rarely associated congenital laryngeal anomaly, as the presenting feature.

**Case presentation:**

A four-day old, full-term, male infant born via uncomplicated vaginal delivery with a nursery course significant for failed hearing screen presented to an emergency department (ED) with respiratory distress and worsening stridor. He was transferred to a level III neonatal intensive care unit (NICU) for further evaluation and required intubation due to progressive hypercarbia. Laryngoscopy revealed left-sided unilateral vocal fold paralysis (VFP). He underwent further evaluation that included a normal MRI brain, neck and chest. Genetics was consulted with concern for dysmorphic features on physical exam. Following gene panel testing, VFP was attributed to known association with CHARGE syndrome. Airway edema was noted on laryngoscopy that prevented extubation until two months of age. Further features of CHARGE syndrome identified included colobomas, glaucoma, sensorineural hearing loss, and genital abnormalities. He was discharged in room air and following gastrostomy tube placement with otolaryngology follow up.

**Conclusions:**

Although choanal abnormalities are classically associated with CHARGE syndrome, other upper airway anomalies such as VFP may be present. VFP is a rarely reported anomaly in association with CHARGE syndrome (Naito Y, Higuchi M, Koinuma G, Aramaki M, Takahashi T, Kosaki K. Upper airway obstruction in neonates and infants with CHARGE syndrome. Am J Med Genet 2007;143A:1815–20; Morgan D, Bailey M, Phelps P, Bellman S, Grace A, Wyse R. Ear-nose-throat abnormalities in the CHARGE association. Arch Otolaryngol Head Neck Surg 1993;119:49–54).

## Introduction

CHARGE syndrome is an autosomal dominant disorder that most often results from *de novo* mutations in the *CHD7* (chromodomain helicase DNA binding protein 7) gene. The acronym CHARGE includes coloboma, heart defect, atresia choanae, retarded growth and development, genital hypoplasia, and ear anomalies/deafness. Choanal abnormalities are present in 50–60 % of patients [1]. Other upper airway anomalies associated with CHARGE syndrome include tracheomalacia, tracheoesophageal fistula, laryngomalacia, subglottic stenosis, and laryngeal clefts. [2, 3] Association with vocal fold paralysis (VFP) has been reported in the literature, but only rarely; for example, in one large cohort of 50 patients with CHARGE syndrome, just one patient was found to have VFP which developed following iatrogenic injury attributed to cardiac surgery [3]. No detailed case reports of congenital VFP associated with CHARGE syndrome are included in the literature.

## Case presentation

A four-day old male infant born at 39 4/7 weeks’ gestation presents to an emergency department (ED) with worsening respiratory distress and stridor exacerbated by feeding and supine positioning.

He was born to a 31-year-old G2P2 mother with serologies significant for positive indirect Coomb’s test via an uncomplicated vaginal delivery with APGARs of eight and nine at one and 5 min, respectively. His birth weight was 3,050 *g*. Fetal ultrasound demonstrated concern for intracardiac focus, with an otherwise uncomplicated pregnancy. Nursery course was significant only for a failed hearing screen bilaterally and normal echocardiogram. The infant was discharged home on day of life two. He was noted to have nasal congestion at time of discharge. Family was instructed to suction his nose and use saline drops as needed.

On day of life four, the infant’s mother noted stridor associated with difficulty feeding and is directed to the ED by the pediatrician. Examination in the ED is significant for high-pitched, inspiratory stridor with tracheal tugging and subcostal retractions. Additionally, he is noted to have pectus excavatum. The stridor and increased work of breathing improve with prone or side lying positioning. Laboratory evaluation is significant for respiratory acidosis on venous blood gas [pH 7.18 and CO2 73 mmHg (9,732 Pa)] and urinalysis that reveals moderate bacteria and 11–20 white blood cells without the presence of urine nitrite or leukocyte esterase. A complete blood count is reassuring. He is placed on high flow nasal cannula and initiated on ampicillin and gentamicin after obtaining blood cultures.

He is escalated to continuous positive airway pressure (CPAP) and transferred to a level III neonatal intensive care unit (NICU) for further management and otolaryngology evaluation. A nasogastric tube was passed through bilateral nares without difficulty. Flexible laryngoscopy showed an immobile left vocal cord and narrow nasal cavities with edema of nasal turbinates, bilateral arytenoids, and vocal cords. Bronchoscopy did not reveal any further abnormalities. Further physical exam was notable for subtle dysmorphic features including low set ears, retromicrognathia, microphthalmia, and clinodactyly of toes. Empiric antibiotics were discontinued following reassuring infectious work-up. A multidisciplinary team was consulted.

The patient eventually required intubation at two weeks of life due to worsening respiratory failure. Nasal Ciprofloxacin and Dexamethasone were administered for upper airway edema. An MRI of the brain, neck, and chest showed a normal course of the recurrent laryngeal nerves. Genetics evaluation included a normal chromosomal microarray analysis (CMA). Further gene panel testing was positive for pathogenic variant in *CHD7* gene, consistent with CHARGE syndrome. In this case, VFP was attributed to known association with CHARGE syndrome. Continued evaluation revealed further features consistent with CHARGE syndrome including bilateral colobomas, glaucoma, confirmed hearing loss, and genital abnormalities.

Repeat bronchoscopy on day of life 42 demonstrated improvement in overall laryngeal edema but continued mild edema of bilateral vocal cords. The patient was extubated post-bronchoscopy in the setting of improved airway edema. However, he was subsequently reintubated on day of life 46 due to worsening respiratory failure. Repeat laryngoscopy that day had shown continued edema. At this point, the patient had already received multiple short courses of Dexamethasone in an attempt to decrease the airway edema. Due to the need for reintubation, he was treated with topical Tobramycin and Dexamethasone applied directly to vocal cords via direct laryngoscopy for five days. Following this treatment, he was successfully extubated at two months old and underwent gastrostomy tube placement at three months.

At the time of gastrotomy tube placement, he underwent an additional bronchoscopy and microlaryngoscopy which demonstrated the originally affected left vocal cord was fully mobile but showed new immobility of right cord; the etiology of this is unclear. Both vocal folds were mildly edematous still but much improved from prior evaluation. Patient was discharged in room air at age four months and continues to follow closely with otolaryngology. On repeat outpatient laryngoscopy at four months of age, both cords appeared mobile with continued airway edema.

## Discussion

Neonatal stridor is a relatively common symptom in general pediatrics and emergency medicine settings. Laryngomalacia accounts for 70 % of cases of neonatal stridor [4]; however, it is important to distinguish other causes. Important features in the history include severity, progression, association with feeding or sleeping difficulties, and any cyanotic or apneic events [5]. Additional key history includes birth history and any history of intubation, surgery, or procedures. Passage of a flexible catheter through each nasal passage may be performed to exclude choanal atresia, as stertor may be easily confused with stridor.

Stridor is produced from the larynx or trachea. Although exceptions are possible, inspiratory stridor is most often associated with supraglottic conditions, biphasic stridor with glottic or subglottic conditions, and expiratory stridor with tracheal conditions. There are a variety of laryngeal and tracheal conditions to consider (Table 1).

**Table 1:** Differential of neonatal stridor.

Characterization of stridor	Anatomic location	Differential diagnoses
Inspiratory	Supraglottic	Laryngomalacia
		Congenital cysts
		Congenital high airway obstruction syndrome
Biphasic	Glottic	Vocal fold paralysis
		Laryngeal web
		Laryngeal atresia
		Laryngeal cleft
	Subglottic	Subglottic stenosis
		Subglottic hemangioma
Expiratory	Trachea	Primary tracheomalacia
		Secondary tracheomalacia
		Tracheal stenosis

VFP is the second most common congenital anomaly of the larynx secondary to laryngomalacia [4]. A key feature that can assist in distinguishing VFP from other etiologies of neonatal stridor is the presence of a hoarse or breathy cry, the manifestation of dysphonia in infants [5]. The stridor of unilateral VFP is usually mild or may not be present as compared to bilateral paralysis that typically requires emergent airway intervention with pronounced stridor. Conversely, dysphonia is usually more pronounced in unilateral paralysis. The overall incidence of VFP has not been determined but is more common in preterm infants [6]. Various syndromes have been found to be associated including CHARGE, trisomy 21, Robinow syndrome, cerebro-oculofacioskeletal syndrome, Moebius syndrome, 22q11 deletion syndrome, Pierre-Robin sequence and Charcot Marie Tooth disease.

Broad categories of VFP causes include central nervous system lesions, iatrogenic injury, and mediastinal or chest anomalies. Iatrogenic causes are most common [7]. Knowledge of anatomy aids in understanding the various causes of VFP. The recurrent laryngeal nerve (RLN) supplies both motor and sensory innervation to the vocal folds. The RLN is a branch of the vagus nerve (cranial nerve X), which originates from the nucleus ambiguous in the brainstem. The course of the left RLN is longer as compared to the right RLN, coursing along the ductus arteriosus and aortic arch. Consequently, the left RLN is more prone to injury. Cardiac procedures, particularly patent ductus arteriosus ligation and other aortic arch repairs, have a high incidence of post-operative VFP. Other potential iatrogenic causes include extracorporeal membrane oxygenation cannulation and birth trauma. The use of vacuum or forceps in delivery and breech presentation increase this risk [6]. Any intracranial pathology resulting in increased pressure on the vagus nerve should also be considered. For instance, a case report described bilateral VFP as the only presenting feature of Chiari malformation [8]. Hypoxic ischemic encephalopathy and intracranial hemorrhage are other important considerations with case reports describing these in association with VFP as well [9, 10]. If no underlying cause is identified, VFP is then classified as idiopathic.

Flexible laryngoscopy is the gold standard to diagnose VFP. Computed tomography (CT) or magnetic resonance imaging (MRI) should be performed if the cause is unclear. Importantly, images need to include brain, neck, and chest to evaluate the trajectory of the RLN in addition to structural anomalies of the airway.

The spontaneous rate of recovery for all VFP is 48–62 % [4]. Tracheostomy is necessary in 54–69 % of cases and is more likely to be necessary in bilateral paralysis [6]. If intubation is necessary, a period of observation prior to tracheostomy is often appropriate to monitor for spontaneous improvement. Procedures that otolaryngology may consider for severe VFP include endoscopic anterior and posterior cricoid split, laryngeal reinnervation, suture lateralization, carbon dioxide (CO2) laser posterior transverse partial cordectomy, CO2 laser arytenoidectomy, and arytenoidectomy with suture lateralization [4]. In infants with unilateral VFP, injection laryngoplasty with a resorbable material can improve oral feeding and phonation. As children with unilateral VFP become older, laryngeal reinnervation and thyroplasty may be considered [6]. Prior to the initiation of oral feeds, a swallow evaluation is critical due to the high incidence of comorbid dysphagia and risk for aspiration. A large study demonstrated that 40.8 % of patients with VFP required gastrostomy tube [7]. Close outpatient follow up with otolaryngology and other subspecialties as needed is warranted.

Our case is illustrative of the wide differential diagnosis for neonatal stridor and significant morbidity associated with VFP. Our patient’s VFP diagnosis prompted further evaluation leading to the diagnosis of multiple other congenital abnormalities with an ultimate diagnosis of CHARGE syndrome. This case also illustrates that other upper airway anomalies, aside from choanal atresia, may be found in patients with CHARGE syndrome, including VFP. Given the successful extubation of our patient following treatment with intranasal Ciprofloxacin and Dexamethasone and laryngeal application of Tobramycin and Dexamethasone ointment, these treatments may be considered for similar cases.
